# Reliability of Hamilton-Norwood Classification

**DOI:** 10.4103/0974-7753.58554

**Published:** 2009

**Authors:** M Guarrera, P Cardo, P Arrigo, A Rebora

**Affiliations:** Department of Endocrinological and Medical Sciences, Section of Dermatology, Genoa, Italy; Department of Health Sciences, Section of Dermatology, University of Genoa, Genoa, Italy; CNR, Institute for Macromolecular Studies, Genoa, Italy

**Keywords:** Alopecia, baldness, hair, Hamilton

## Abstract

**Background:**

Hamilton-Norwood scale (HNS) has been largely used to assess clinically the severity of androgenetic alopecia (AGA), especially for therapeutical trials and even to establish its association with important diseases such as ischemic heart disease and prostate cancer.

**Objective:**

To study HNS reproducibility in the hands of dermatologists and dermatology residents.

**Materials and Methods:**

Seven dermatologists and 16 residents in dermatology classified 43 photographs of male heads with different degrees of AGA. In a second study, 8 appraisers (3 dermatologists and 5 residents in dermatology) examined 56 pictures with the same procedure and repeated the observation 3 months later. In the first study, the inter-rater agreement was estimated by calculating an intra-class correlation coefficient (ICC). In the second study, for intra-rater repeatability, each rater's scores from session 1 were paired with his/her scores for the same subjects in session 2, and the ordinary least products linear regression was calculated.

**Results:**

In the first study, the concordance of appraisers was unsatisfactory (ICC = 0.63-0.68)]. In the second study, repeatability was poor, without any significant difference between dermatologists and dermatology residents.

**Comment:**

Reliability of HNS is unsatisfactory even in the hands of expert appraisers. To obtain better reliability, the number of classes should be reduced, but with such reduction HNS would be usable to classify patients only in a broad way.

## INTRODUCTION

Hamilton-Norwood scale (HNS) has been largely used to assess clinically the severity of androgenetic alopecia (AGA), especially for therapeutical trials, and to establish its association with important diseases such as ischemic heart disease and prostate cancer.[1‐8] Its reproducibility, however, has been poorly studied.[9,10] We investigated HNS reproducibility in the hands of dermatologists and dermatology residents and found it unsatisfactory.

## MATERIALS AND METHODS

Seven dermatologists and 16 dermatology residents were recruited as raters. They classified 43 photographs of male heads showing different degrees of AGA by constantly checking a cartoon depicting HNS, except the anterior model. Each examiner independently scored the pattern of each photograph as I, II, III, III vertex, IV, V, VI or VII degree using ordinals between 1 and 7 plus a value of 3.5 corresponding to III vertex degree.

In a second study, 3 dermatologists and 5 dermatology residents scored 56 randomly selected photographs on two separate occasions, 3 months apart, with the same procedure. The pictures were shown in the second session in the same order as in the former one.

Statistical analyses were done using SPSS version 17.0 (SPSS Inc., Chicago, IL).

In the first study, the inter-rater reproducibility was estimated by calculating the intra-class correlation coefficient (ICC) based on an ANOVA mixed model.[11] The value of ICC tends to be smaller than 1. The closer the ICC is to 1, the more similar the samples are. In the second study for intra-rater repeatability, each rater's scores from session 1 were paired with his/her scores for the same subjects in session 2, and the ordinary least products (OLP) linear regression and R^2^ were calculated.[12] R^2^ is a measure of linear association between two variables and corresponds to 1 when correlation is perfect.

## RESULTS

In the first study, data were greatly dispersed (coefficients of variation varied from 66% to 357%). ICC was 0.65 (*P* < 0.001). Only a slight difference between dermatologists and dermatology residents (0.631 *vs*. 0.683, respectively) was observed [Table 1].

**Table 1:** Intraclass correlation coefficients

Raters	N	Intraclass correlation	95% confidence interval
Residents	16	0.631	0.527 ÷ 0.739
Dermatologists	7	0.683	0.571 ÷ 0.788
Total	23	0.655	0.556 ÷ 0.757

Two-way random effects model where both raters' effects and measure effects are random, using an absolute agreement definition

In the second study, the correlation between the data from the first session and those from the second one (repeatability) was unsatisfactory. In fact, only one dermatologist achieved 0.75 of adjusted R^2^. No significant difference between dermatologists and dermatology residents was observed [Table 2].

**Table 2:** Ordinary least products linear regression analysis for repeatability

Raters	Name	Slope	Bias	Adjusted R^2^
	GV	0.893	-0.145	0.6363
	AV	0.845	0.822	0.7443
Residents	MR	0.919	0.405	0.7111
	AC	0.760	0.887	0.4578
	MB	0.788	0.970	0.7226
	LY	0.880	0.687	0.6887
Dermatologists	FV	0.915	0.308	0.5991
	MG	0.856	0.480	0.7535

In a perfect repeatibility, slope must be 1 and bias 0. R2 varies from 0 to 1.

## DISCUSSION

AGA severity and drug efficacy have been repeatedly assessed by HNS. HNS has been employed even to evaluate issues as important as the relationship of baldness with coronary artery disease and prostate cancer. In such cases, the assessment has often been done by non-dermatologists or even by the patients themselves. HNS reliability has been verified recently. Taylor and - colleagues[9] examined 105 males who were invited to select a picture that best represented their balding pattern. Two trained appraisers independently assessed the participants' balding patterns, and the men's self-assessment was compared with the assessment of the two trained appraisers. Appraisers were very reliable in their assessment of balding pattern (Cohen's κ = 0.83); but when compared to the two trained appraisers, their concordance fell to 0.39-0.46. It should be noted, however, that the classes of HNS were reduced to 4. In a second analysis, Taylor *et al*. studied the concordance between the assessment by each observer and that by the patients of the balding patterns of the patients at age 35. The latter proved to be only moderately accurate. Littmann and -colleagues[10] studied 100 men who were invited to describe their hair patterning at age 30, at age 45 and their current age (50 to 76). κ values were 0.74, 0.71 and 0.81, respectively, but HNS classes were reduced to only 3. In addition, κ for the comparison of the subjects' report with the interviewer's assessment was only 0.47.

Two considerations should be done, however, when appraising those studies. The first is that to achieve a good agreement, HNS classes had to be reduced to 4 and 3, respectively.[9,10] The second consideration concerns the applicability of Cohen's κ and Fleiss' κ statistics. Fleiss' κ is a statistical measure of inter-rater reliability, which, differently from Cohen's κ, works for multiple raters giving categorical ratings to a fixed number of items. It expresses the extent to which the observed agreement among raters exceeds that expected if all raters did their ratings completely randomly. This test ranks the agreement on the base of estimated k value. As currently accepted, the agreement is regarded as poor if κ ≤ 0, slight if 0 < κ ≤ 0.20, fair if 0.20 < κ ≤ 0.40, moderate if 0.40 < κ ≤ 0.60, substantial if 0.60 < κ ≤ 0.80, perfect if 0.80 < κ ≤ 1.00.[13] One disadvantage of Fleiss' k is that it ignores any ordering. For example, the disagreement between class III and class VII has the same weight as that between class III and class III vertex.[14] We adopted ICC just to overcome such problem.

On the other hand, if to obtain a good reliability the number of classes is reduced, the classification becomes usable, as Chamberlain and Dawber put it, only in the broader classification of patients who are likely to respond to therapies.[15]

ICC is a descriptive statistic that can be used when quantitative measurements are made on units that are organized into groups. It describes how strongly units in the same group resemble each other and is currently used to assess the reproducibility of quantitative measurements made by different observers measuring the same quantity.

Assessing AGA severity is not easy, especially if reliable, inexpensive and minimally invasive means are required. In our hands, HNS proved unsatisfactorily reproducible. A new classification, universal for men and women, has been recently introduced[16] but still awaits validation. In its stead, computer-assisted measurements of hair density[17] or a cheaper and easier method as the modified wash test[18] should be adopted. The latter provides the percentage of vellus telogen hairs, which is probably a more accurate measure of AGA severity.
